# A Spiking Working Memory Model Based on Hebbian Short-Term Potentiation

**DOI:** 10.1523/JNEUROSCI.1989-16.2016

**Published:** 2017-01-04

**Authors:** Florian Fiebig, Anders Lansner

**Affiliations:** ^1^Lansner Laboratory, Department of Computational Science and Technology, Royal Institute of Technology, 10044 Stockholm, Sweden,; ^2^Department of Numerical Analysis and Computer Science, Stockholm University, 10691 Stockholm, Sweden, and; ^3^Institute for Adaptive and Neural Computation, Edinburgh University, EH8 9AB Edinburgh, Scotland

**Keywords:** Hebbian plasticity, primacy, recency, short-term potentiation, word list learning, working memory

## Abstract

A dominant theory of working memory (WM), referred to as the persistent activity hypothesis, holds that recurrently connected neural networks, presumably located in the prefrontal cortex, encode and maintain WM memory items through sustained elevated activity. Reexamination of experimental data has shown that prefrontal cortex activity in single units during delay periods is much more variable than predicted by such a theory and associated computational models. Alternative models of WM maintenance based on synaptic plasticity, such as short-term nonassociative (non-Hebbian) synaptic facilitation, have been suggested but cannot account for encoding of novel associations. Here we test the hypothesis that a recently identified fast-expressing form of Hebbian synaptic plasticity (associative short-term potentiation) is a possible mechanism for WM encoding and maintenance. Our simulations using a spiking neural network model of cortex reproduce a range of cognitive memory effects in the classical multi-item WM task of encoding and immediate free recall of word lists. Memory reactivation in the model occurs in discrete oscillatory bursts rather than as sustained activity. We relate dynamic network activity as well as key synaptic characteristics to electrophysiological measurements. Our findings support the hypothesis that fast Hebbian short-term potentiation is a key WM mechanism.

**SIGNIFICANCE STATEMENT** Working memory (WM) is a key component of cognition. Hypotheses about the neural mechanism behind WM are currently under revision. Reflecting recent findings of fast Hebbian synaptic plasticity in cortex, we test whether a cortical spiking neural network model with such a mechanism can learn a multi-item WM task (word list learning). We show that our model can reproduce human cognitive phenomena and achieve comparable memory performance in both free and cued recall while being simultaneously compatible with experimental data on structure, connectivity, and neurophysiology of the underlying cortical tissue. These findings are directly relevant to the ongoing paradigm shift in the WM field.

## Introduction

Working memory (WM) is a key component of cognition. It maintains information over seconds and minutes in a form that allows animals to act beyond the here and now. WM is updated by selectively attended external information and activated long-term memory representations. Mammalian prefrontal cortex (PFC) is generally believed to play a key role in WM (Fuster, 2009; D'Esposito and Postle, 2015).

The most common theory about the neural mechanisms of WM is that of persistent elevated activity in a recurrently connected neural network, presumably located in the PFC (Funahashi et al., 1989; Goldman-Rakic, 1995; Tsakanikas and Relkin, 2007). This theory was implemented in early spiking neural network models of persistent activity WM (Camperi and Wang, 1998; Compte et al., 2000). However, recent reexamination of experimental data has shown that PFC activity in single units during delay periods is much more variable than predicted by such a theory and associated computational models (Shafi et al., 2007). Contrary to predictions from the theory, memory may not be abolished by pauses in the elevated activity (LaRocque et al., 2013; Stokes, 2015) and recent experiments link multi-item WM information to discrete γ burst events rather than persistent activity (Honkanen et al., 2015; Lundqvist et al., 2016).

Hypotheses about neural mechanisms behind WM are thus currently under revision (Barak and Tsodyks, 2014; Sreenivasan et al., 2014; D'Esposito and Postle, 2015; Eriksson et al., 2015; Stokes, 2015) and alternative models based on synaptic plasticity have been suggested (Sandberg et al., 2003; Mongillo et al., 2008; Lundqvist et al., 2011). Many of these are based on short-term nonassociative (non-Hebbian) synaptic facilitation that can buffer a memory in time (Zucker and Regehr, 2002; Mongillo et al., 2008), replacing strict persistency. Periodic attractor reactivations may repeatedly refresh decaying synaptic facilitation, thus retaining memory.

Facilitation-based WM models have a severe shortcoming: they are unable to explain encoding of novel associations. Their learning mechanisms are presynaptic in nature, implying that all outgoing synapses from an active neuron will be enhanced. Indeed, non-Hebbian plasticity can only bring online already existing representations (i.e., synaptic structures preshaped earlier via Hebbian LTP) (Durstewitz et al., 2000).

Recently, different forms of early and fast expressing Hebbian forms of synaptic plasticity (e.g., short-term potentiation [STP]) have been characterized experimentally and proposed as candidates for synaptic WM (Erickson et al., 2010; Park et al., 2014). STP is expressed after brief high-frequency bursts and remarkably decays not in a time-, but activity-dependent, manner (Volianskis et al., 2015).

Given the fundamental importance of WM processes, difficulties to find alternative explanations, and the emergence of experimental evidence on STP, we find it well worth examining the hypothesis of STP as a mechanism for WM. We do this using a spiking attractor network model of cortex, which exhibits basic cortical operations, such as associative memory, pattern completion, and rivalry (Lansner, 2009). We build on a previously published such model, which demonstrated how facilitation-based reactivations in a cortical microcircuit with fast, basket-cell mediated feedback-inhibition can successfully reproduce brief, narrow γ-band bursts, linked to multi-item memory activity in nonhuman primate PFC (Lundqvist et al., 2011, 2016). We further extended this model with fast Hebbian synaptic plasticity in line with previous work on a nonspiking network model of WM (Sandberg et al., 2003).

We focus on a multi-item WM task of encoding and immediate recall of a word list, which is a standard neuropsychological paradigm that has also previously been studied in a nonspiking neural network model (Lansner et al., 2013). We demonstrate known cognitive phenomena, such as primacy and recency, at human level memory performance in both free and cued recall. We compare our model with electrophysiological data from cortex, such as PSP (EPSP, IPSP) amplitudes resulting from memory encoding, and draw parallels to very recent electrophysiological recordings of multi-item WM in nonhuman primates (Lundqvist et al., 2016).

## Materials and Methods

Here we present the architecture of the spiking neural network model, as well as neuron and synapse models, including synaptic plasticity rules. We use the NEST simulator version 2.2 (Gewaltig and Diesmann, 2007) for our simulations. Code is available upon request. A detailed listing of model parameters and values can be found in Tables 1 (network model and connectivity), 2 (neural and synaptic parameters), and 3 (stimulation and recall testing).

**Table 1.** Network layout and connectivity*^a^*

Cortical patch size	2.88 × 2.16 mm	PP connection probability (excluding autapses)	*p_PP_*	0.2
Simulated HCs	*n_HC_* 16	PB connection probability	*p_PB_*	0.7
Simulated MCs	*n_MC_* 192	PB connection conductance	*g_PB_^AMPA^*	3.5 nS
No. of patterns	*n_a_* 12	BP connection probability	*p_BP_*	0.7
MC grid size	16 × 12	BP connection conductance	*g_BP_^GABA^*	−40 nS

*^a^*P, Pyramidal cell; B, basket cell.

**Table 2.** Neural, synaptic, and BCPNN parameters

Adaptation current	b	86 pA	Utilization factor	U	0.25	BCPNN AMPA gain	*w_gain_^NMDA^*	6.62 nS
Adaptation time constant	τ*_w_*	500 ms	Depression time constant	τ*_rec_*	500 ms	BCPNN NMDA gain	*w_gain_^NMDA^*	0.58 nS
Membrane capacitance	*C_m_*	280 pF	AMPA synaptic time constant	τ*^AMPA^*	5 ms	BCPNN bias current gain	β*_gain_*	65 pA
Leak reversal potential	*E_L_*	−70 mV	NMDA synaptic time constant	τ*^NMDA^*	150 ms	BCPNN lowest spiking rate	*f_min_*	0.2 Hz
Leak conductance	*g_L_*	14 pS	GABA synaptic time constant	τ*^GABA^*	5 ms	BCPNN highest spiking rate	*f_max_*	20 Hz
Upstroke slope factor	Δ*_T_*	3 mV	AMPA reversal potential	*E^AMPA^*	0 mV	BCPNN lowest probability	ε	0.01
Spike threshold	*V_t_*	−55 mV	NMDA reversal potential	*E^NMDA^*	0 mV	BCPNN Spike event duration	Δ*t*	1 ms
Spike reset potential	*V_r_*	−80 mV	GABA reversal potential	*E^GABA^*	−75 mV	P trace time constant	τ*_p_*	5 s

**Table 3.** Stimulation protocol and recall testing parameters

Background activity rate	*r_bg_*	750 Hz	Free recall time (Study A)	*t_free_^Study A^*	45 s
Alternative background rate	*r_bg_^Demo2^*	570 Hz	Free recall time (Study B)	*t_free_^Study B^*	30 s
Excitatory background conductance	*g_bg_^exc^*	1.5 nS	Cued recall time (per cue)	*t_cued_^Study B^*	5 s
Inhibitory background conductance	*g_bg_^inh^*	−1.5 nS	Attractor detection threshold	*r_thresh_*	10 Hz
Interstimulus interval	*T_stim_^StudyA,B^*	1 s	Cue stimulation length	*t_stim_^cue^*	20 ms
Interstimulus interval	*T_stim_^Demo2^*	0.5 s	Cue stimulation rate	*r_stim_^cue^*	850 Hz
Stimulation duration	*t_stim_*	1 s	Attractor detection threshold	*r_thresh_*	10 Hz
Stimulation rate	*r_stim_*	1.7 kHz	Pattern stimulation conductance	*g^stim^*	1.5 nS

### 

#### Network model

The computational network model used here is inspired by cortical microcircuit architecture principally follows previous models (Lundqvist et al., 2006; Tully et al., 2016) and is best understood as an abstraction of a subsampled associative cortical layer 2/3 network.

The network follows a columnar organization of neocortex (Hubel and Wiesel, 1977; Mountcastle, 1997) and consists of *n_HC_* = 16 hypercolumns (HC_0_-HC_15_) that contain a total of 5760 pyramidal cells and 384 inhibitory basket cells. Each HC contains 24 basket cells, and its pyramidal cell population can be further divided into 12 functional minicolumns (MCs) consisting of 30 pyramidal neurons each. This constitutes a downsampling from ∼100 MC per HC in cortex, whereas 30 pyramidal neurons per MC represent approximately the layer 2/3 population of an MC. In further discussion, we use the terms local and global to denote whether something belongs to a HC (e.g., elements and processes of a specific MC) or to the larger network as a whole.

**Figure 1.**
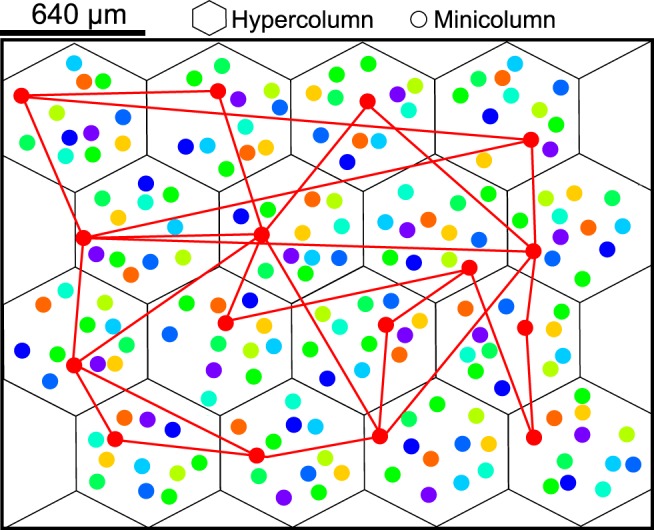
Schematic of the model layer 2/3 network. The network is comprised of 16 HCs, spanning a 2.88 mm × 2.16 mm patch of neocortex. Each HC contains 12 (differently colored) MCs, which are composed of 30 pyramidal cells each and preferentially active for 1 of 12 stimulated activity patterns. For one of these patterns (red), we also indicate some of the sparse long-range excitatory connections between subsampled pyramidal cells of similar selectivity that emerges after learning. Colors were chosen to be consistent with a 12-item memory model presented later.

HCs are laid out on a hexagonal grid corresponding to a subsampled 2.88 mm × 2.16 mm patch of neocortex (Fig. 1). Each of the nonoverlapping HCs has a diameter of ∼640 μm, comparable with estimates of cortical column size (Mountcastle, 1997). We computed axonal delays *t_ij_* between presynaptic neuron *i* and postsynaptic neuron *j*, based on a conduction velocity *V* of 0.2 mm/ms and the Euclidean distance between respective MCs. Conduction delays were randomly drawn from a normal distribution with mean according to the connection distance and a relative SD of 15% of the mean to account for individual arborization differences. Further, a minimal conduction delay of 1 ms was added to reflect not directly modeled delays, such as diffusion of transmitter over the synaptic cleft, dendritic branching, thickness of the cortical sheet, and the spatial extent of MCs as follows:

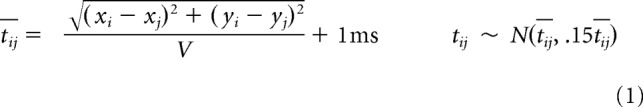


#### Connectivity

Pyramidal neurons project laterally to basket cells within their own HC via AMPA-mediated excitatory projections with a connection probability of p_PB_ (i.e., connections are randomly drawn without duplicates until such a target fraction of all possible pre-post connections is reached). In turn, they receive GABAergic feedback inhibition from basket cells (p_BP_). This loop of strong connections implements a competitive soft-WTA subnetwork within each HC (Douglas and Martin, 2004). Pyramidal cells form AMPA- and NMDA-mediated connections both within and across HCs at connection probability p_PP_. These projections are implemented as plastic synapses, as explained in Spike-based BCPNN learning rule. The model thus features a total of 13.3 million plastic AMPA- and NMDA-mediated connections between pyramidal cells, as well as ∼100,000 excitatory connections from pyramidal cells to basket cells in their respective HC and an equal number of inhibitory connections back to their respective pyramidal cell populations.

#### Neuron model

We use an AdEx IAF neuron model with spike-frequency adaptation (Brette and Gerstner, 2005) that was modified recently (Tully et al., 2014) for compatibility with a custom-made BCPNN synapse model in NEST through the addition of the intrinsic excitability current *I*_β*_j_*_ (see Spike-based Bayesian learning rule). The model was simplified by excluding the subthreshold adaptation dynamics. Membrane potential *V_m_* and adaptation current are described by the following equations:

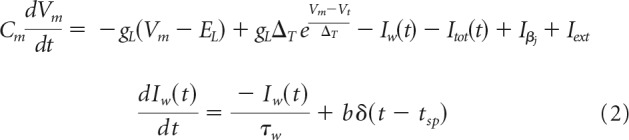
 The membrane voltage changes through incoming currents over the membrane capacitance *C*_m_. A leak reversal potential *E_L_* drives a leak current through the conductance *g_L_*, and an upstroke slope factor Δ*_T_* determines the sharpness of the spike threshold *V_t_*. Spikes are followed by a reset to *V_r_*. Each spike increments the adaptation current by *b*, which decays with time constant τ*_w_*. Basket cells connect via static synapses rather than BCPNN synapses, and they feature neither an intrinsic excitability current *I*_β*_j_*_ nor spike-triggered adaptation. In addition to external input *I_ext_* (see Stimulation protocol), neurons receive a number of different synaptic currents from other presynaptic neurons in the network (AMPA, NMDA, and GABA), which are summed at the membrane according to the following:

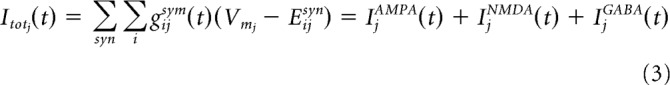


#### Synapse model

Excitatory AMPA and NMDA synapses have a reversal potential *E^AMPA^* = *E^NMDA^*, whereas inhibitory synapses drive the membrane potential toward *E^GABA^*. In addition to BCPNN learning (see Spike-based BCPNN learning rule), plastic synapses are also subject to synaptic depression (vesicle depletion) according to the Tsodyks-Markram formalism (Tsodyks and Markram, 1997) as follows:

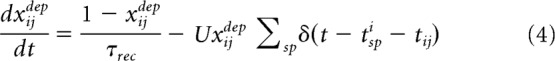
 The fraction of synaptic resources available at each synapse *x_ij_^dep^* is depleted by a synaptic utilization factor (*U*) with each spike transmission and decays with τ*_rec_* back toward its maximum value of 1. Every presynaptic input spike (at *t_sp_^i^* with transmission delay *t_ij_*) thus evokes a transient synaptic current through a change in synaptic conductance that follows an exponential decay with time constants τ*^syn^* depending on the synapse type (τ*^AMPA^* ≪ τ*^NMDA^*).

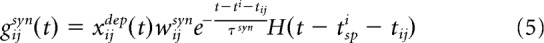

*H*(·) denotes the Heaviside step function, and *w_ij_^syn^* is the peak amplitude of the conductance transient, learned by the following Spike-based BCPNN learning rule.

#### Spike-based BCPNN learning rule

Plastic AMPA and NMDA synapses are modeled with a spike-based version of the Bayesian Confidence Propagation Neural Network (BCPNN) learning rule (Wahlgren and Lansner, 2001; Tully et al., 2014, 2016). For introductory purposes, we only highlight a few key equations here. For a full derivation of the learning rule from Bayes rule, deeper biological motivation, and proof of concept, see Tully et al. (2014). The E trace, which is critical for allowing delayed reward learning, has been omitted because such conditions are not applicable here. This is equivalent to setting the corresponding time constant (τ*_E_*) to a very small value in the complete model.

Briefly, the BCPNN learning rule makes use of biophysically plausible local traces to estimate normalized presynaptic and postsynaptic firing rates (referred to as *p_i_*, and *p_j_* respectively), as well as coactivation (*p_ij_*). As was shown earlier, these *P* traces can be combined to implement Bayesian inference because connection strengths and MC activations have a statistical interpretation (Sandberg et al., 2002; Fiebig and Lansner, 2014; Tully et al., 2014).

Presynaptic and postsynaptic spike trains (*S_i_* and *S_j_*, respectively) are formally described as summed Dirac δ pulses at spike times *t^i^* and *t^j^* as follows:

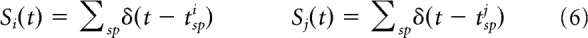
 Two consecutive levels of exponentially weighted moving averages *Z*, and *P* smoothen the spike train. An initial lowpass filter generates presynaptic and postsynaptic traces *Z_i_* and *Z_j_* as follows:

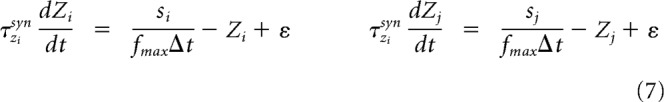

Equation 7 also achieves a linear normalizing transformation between the neuronal spike rate ∈ [*f_min_*, *f_max_*] and the probability space ∈ [ε,1], where ε represents the lowest attainable probability estimate. The *Z* trace of a neuron firing at *f_max_* rate will average to 1, whereas a persistently silent neuron will have a *Z* trace converging on ε. Δ*t* denotes the spike event duration. Presynaptic and postsynaptic time constants τ_*z_i_*_^*syn*^,τ_*z_j_*_^*syn*^ are the same but differ between AMPA and NMDA synapses as follows:

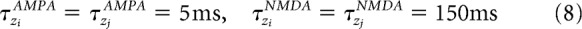
 The larger NMDA time constant reflects the slower closing dynamics of NMDA-receptor gated channels. Experimental findings suggest that NMDA kinetic properties vary 50-fold (40–2000 ms) depending on receptor subtype composition (Paoletti et al., 2013). We choose a value on the slightly higher end of 150 ms for the sake of consistency with a related model (Tully et al., 2016). Activation and coactivation probabilities are estimated, based on filtered *Z* traces as follows:

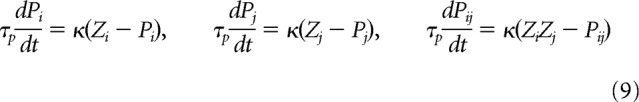
 The parameter κ may reflect the action of endogenous neuromodulators that signal relevance and thus modulate learning efficacy. It can be dynamically modulated; setting κ = 0 can switch off learning and fixate the network. In an effort to highlight the stability of memory networks with spike-based BCPNN learning, we here set κ = 1 throughout all simulation phases. *P* traces constitute memory itself, which decays in a palimpsest fashion. Fast STP decay is known to take place on timescales that are highly variable and activity dependent (Volianskis et al., 2015) (see Experimental support for fast Hebbian synaptic plasticity).

Tully et al. (2014) show that Bayesian inference can be recast and implemented in a network using the spike-based BCPNN learning rule. The prior activation level is here realized as an intrinsic excitability of each postsynaptic neuron, which is derived from the postsynaptic firing rate estimate *p_j_* and implemented in the NEST neural simulator (Gewaltig and Diesmann, 2007) as an individual neural current *I*_β*_j_*_ with scaling constant β_gain_.

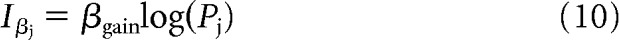

*I*_β*_j_*_ is thus an activity-dependent intrinsic membrane current to the IAF neurons (see Neuron model), similar to the A-type K^+^ channel (Hoffman et al., 1997) or TRP channel (Petersson et al., 2011).

**Figure 2.**
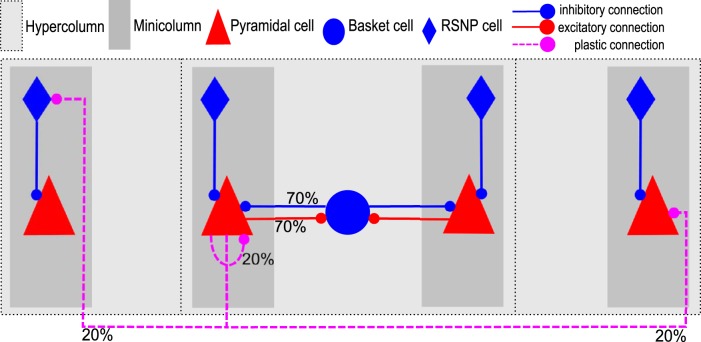
Schematic connectivity of the network model. The probability of a connection from a cell in the presynaptic population to the postsynaptic population is given by the percentages. Learned connections are affected by the spike-based BCPNN learning rule, as described in Spike-based Bayesian learning.

Synaptic weights are modeled as peak amplitudes of the conductance transient (Eq. 5) and determined from the logarithmic BCPNN weight, as derived from the *P* traces with a synaptic scaling constant *w_gain_^syn^*.

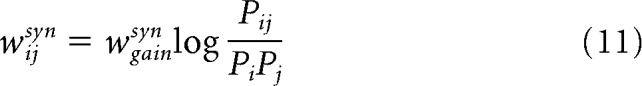
 In our model, AMPA and NMDA synapses make use of *w_gain_^AMPA^* and *w_gain_^NMDA^*, respectively. Their ratio is the AMPA/NMDA amplitude ratio. Experimentally reported values vary considerably (Myme et al., 2003). It has been shown that somewhat lower AMPA/NMDA ratios can be used in a very similar model to learn sequences, as the longer synaptic time constants of the NMDA receptor allow for the learning of temporal correlations between activation patterns (Tully et al., 2016). The logarithm in Equation 11 is motivated by the Bayesian underpinnings of the learning rule, and means that synaptic weights *w_ij_^syn^* multiplex both the learning of excitatory and disynaptic inhibitory interaction. The positive component of *w_ij_^syn^* is here interpreted as the conductance of a monosynaptic excitatory pyramidal to pyramidal synapse (Fig. 2, plastic connection to the MC on the right), whereas the negative component (Fig. 2, plastic connection to the MC on the left) is interpreted as being disynaptic via a dendritic targeting and vertically projecting inhibitory interneuron like a double bouquet and/or bipolar cell (Tucker and Katz, 2003; Kapfer et al., 2007; Ren et al., 2007; Silberberg and Markram, 2007). Such an interneuron would be local to a MC and targeted by several incoming excitatory connections (Lundqvist et al., 2006). Accordingly, all BCPNN connections with a negative weight use a GABAergic reversal potential instead, as in previously published models (Tully et al., 2014, 2016). Model networks with negative synaptic weights have been shown to be functionally equivalent to ones with both excitatory and inhibitory neurons with only positive weights (Parisien et al., 2008). Because of this indirect modeling of inhibition, our network features a lower count of explicitly modeled inhibitory neurons (inhibitory basket cells are modeled explicitly) than would be expected given the common 4:1 E-I ratio.

#### Stimulation protocol

The term *I_ext_* in Equation 2 subsumes specific and unspecific external inputs. To simulate unspecific input from other areas and structures, such as upstream network input from layer 4 and other cortical sources, pyramidal cells are continually stimulated with a zero mean noise background throughout the simulation. Two independent Poisson sources generate spikes at rate *r_bg_*, and connect onto all pyramidal neurons, via nondepressing conductances *g_bg_^exc^* and *g_bg_^inh^*, respectively, which are of equal magnitude and opposite sign. The resulting fluctuations in pyramidal membrane voltages evoke a ground state with low-rate, irregular, asynchronous spiking. Beyond a certain threshold, this can trigger autonomously reactivating attractors in the network.

We implement *n_a_* nonoverlapping training patterns (“attractors”) as conjoint activations of one MC per HC, defined by set 𝕄*_a_* as follows:

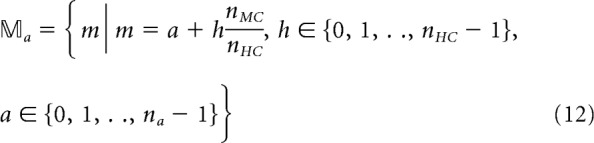
 To train the network on these patterns we drive the pyramidal cells belonging to each of the selected MCs with an additional excitatory Poisson spike train (rate *r_stim_*, length *t_stim_*, and conductance *g^stim^*), such that they fire at ∼20 Hz. Increased firing activates local basket cells, which in turn suppress most of the remaining activation overlap with other patterns through lateral inhibition within each HC. As a result, stimulated patterns are well preserved despite ongoing noisy background activity.

#### Spike train analysis and memory performance metrics

We tracked attractor activity in time by analyzing the firing rate of pattern-specific subpopulations. For each attractor *a* and its component MC_s_*m* ∈ 𝕄_a_, we calculated mean firing rates *r_a_*, respectively, based on the number of pyramidal spikes per member neuron in nonoverlapping 25 ms time bins. To allow for robust detection of attractor states, an attractor *a* was counted as active in time bin *k* if it was sufficiently (Eq. 13), exclusively (Eq. 14), and completely (Eq. 15) active as follows:

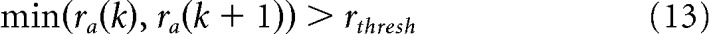


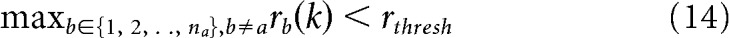


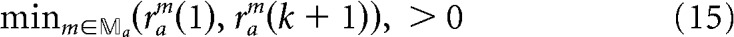
 This implies that the global attractor firing rate needed to sustain a firing rate above a detection threshold for two consecutive time bins (i.e., 50 ms), whereas all stimulus-specific MCs participated with at least one spike, and no other pattern passed this threshold concurrently. The reactivation was then considered continued until one of its conditions (Eqs. 13–15).

We evaluated memory performance through cued and free recall. For the latter, we counted pattern activations for each trained pattern for some time *t_free_*. We detected which patterns activate at least once during free recall and obtained a serial position recall curve by averaging the probability of successful pattern recall over multiple simulated trials. As free recall is a time-dependent process, *t_free_* needs to be long enough, such that the weakest pattern that can be freely recalled will activate at least once. We verified our choice of matching *t_free_* to two experimental memory studies (see Behavioral data comparison) by testing that the serial position recall curve did not change significantly with longer recall time. Furthermore, we analyzed the distribution of pattern transitions (i.e., which patterns activated after one another) to compute a conditional recall probability (Kahana, 1996). It represents the fraction of times that a recalled attractor is followed by another attractor with a certain lag in the study order. Positive lags denote forward transitions in the study order, whereas negative lags denote activation of an earlier pattern. Both the serial position recall curve and conditional recall probability are common measures in cognitive tests of WM and often show a characteristic shape that deviates significantly from respective chance levels, so we compare the model's overall performance against them.

In cued recall, we briefly (*t_stim_^cue^*) stimulated half of each cued pattern *a*, such that only half of the component MCs in M*_a_* became activated. To mimic a loss in saliency, we also cut the pattern stimulation rate (*r_stim_^cue^*) in half, compared with regular training. We then checked whether the pattern activated fully by itself afterward, using the previously described criteria and a recall time *t_cued_* that matched to the experimental study in question.

#### Simulation and parameter search

Simulations were performed on a Cray XC-30 Supercomputer of PDC Centre for High Performance Computing. More than 1 million core-hours were spent on rigorous testing and scanning of the parameter space of the model to ensure that it is robust to parameter variations, and that we fully understand its behavior. The model is tuned primarily toward human cognitive memory performance in word list learning (matching both the timing of experimental protocols and outcomes) and biologically plausible cortex layer 2/3 network parameters. The model is functionally robust and degrades gracefully in case of gradual parameter changes at the chosen operating point. Breakpoints of the qualitative dynamic exist (such as a transition to strictly persistent activity; see Persistent activity) but are generally far away from chosen parameter values.

#### Behavioral data comparison

##### Experimental Study A.

We compared our multi-item WM model to data from the Betula Study (Nilsson et al., 1997), a large prospective cohort study on memory and health. The Betula Study consists of a large battery of cognitive tests, among them a task involving study and immediate free recall of a word list, here referred to as Study A. Participants studied a list of 12 unrelated nouns with the instruction of a free recall test after the final word of the list. Words were presented auditorily at a rate of one item per 2 s, leaving some silence between words. Participants were instructed to recall orally as many words as possible in any order they preferred during a period of 45 s, in keeping with classical studies of free recall (e.g., Murdock, 1962). Participants were counterbalanced against four parallel word lists with mean word frequency of 98 per million words (range 50–200). There were four different conditions with respect to the attentional demands in this task. The data used here were from the condition with focused attention at both study and test. We selected one sample of the Betula Study, consisting of 500 subjects in the age range of 35–55 years with an average of 45 years, tested for the first time in 1988–1990. Participants diagnosed as demented were excluded by following a well-established procedure. For details on the experimental data points used in Figure 7*a*, *b*, see Lansner et al. (2013).

##### Experimental Study B.

For a look at cued recall, we also compared the behavior of our model with data from Gershberg and Shimamura (1994), here referred to as Study B. English-speaking subjects studied lists of 12 common nouns with mean word frequency of 67 per million at a presentation speed of 2 s per word. After learning, different subgroups were asked to either freely recall the items over the course of 30 s, or to complete words based on two- to three-letter word stems from the first or the second half of each word. Subjects were given 5 s to complete each word. Stems were chosen from either the studied list or a second unstudied list, which was used to estimate a guessing baseline, as all words could be completed by at least 10 different English words but only one of the words on the lists. For details on the experimental data points used in Figure 7*c*, *d*, see Gershberg and Shimamura (1994, Experiment 1, their Figs. 1, 2). The original differentiation between performances on forward- and backward-completions of word cues in the experimental study was dropped for the simple comparison with our simulation model, which does not have directionality in the learned memory item/pattern composition.

## Results

In the following, we show three brief introductory demonstrations of the model's basic functionality (Demos 1–3), whereafter we highlight results of two simulation studies with our implementation of Hebbian STP. First, Demo 1 implements a simple single-item memory task that shows functional encoding, maintenance, and recall in the model. In Demo 2, we show how the network can learn and simultaneously store larger numbers of items. Demo 3 examines PSPs underlying successful attractor memory operation in our model. Finally, in Simulation Studies 1 and 2, we show the full dynamics and learning outcomes, replicating results of two typical human word list learning experiments.

### Demo 1: single-item memory encoding and free recall

The most common experimental paradigm investigating persistent activity is the delayed match to sample task, where a single item needs to be held in memory. Typically, PFC cells are recorded, sorted by item selectivity, and filtered according to their temporal activity profile to find cells that are evident neurophysiological correlates of the WM engram. We ask whether a cortical attractor model based on Hebbian STP can capture that task, but also explain how one might arrive at cell activity profiles that show item-specific, seemingly persistent increases in firing rate over the duration of the memory maintenance period of only a few Hertz (see Persistent activity). A new and important aspect of such a demonstration is to also ask, how a persistent activity signal could be understood to be simultaneously compatible with recent critical reviews of the persistent activity hypothesis (Shafi et al., 2007; Sreenivasan et al., 2014; Stokes, 2015) and experimental findings of discrete oscillatory bursts, linked to WM activity in nonhuman primate PFC recordings (Lundqvist et al., 2016).

**Figure 3.**
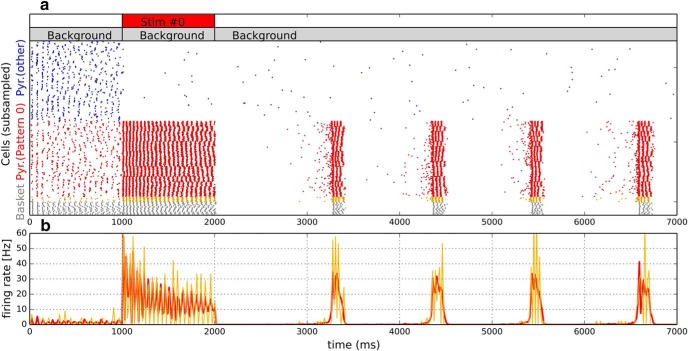
Delayed free recall with one item. For the first second, the network is subject only to uncorrelated background activity, inducing a ground-state baseline firing of 1.3 Hz in the pyramidal cell population. From 1–2 s, we additionally stimulate a subset of neurons (red, indicated by a bar on top of the spike raster), activating one subpopulation (MC) per HC. After stimulation offset, the network runs freely, driven only by uncorrelated background activity, enabling it to reactivate the stored memory and maintain it despite ongoing learning. ***a***, Spike raster of basket cells in gray (subgrouped by HC), pyramidal cells belonging to stimulated population in orange and red, where the orange cells are stimulus-specific cells subsampled from the first HC, and the red cells are all other cells belonging to the stimulated pyramidal population (subgrouped by HC). Some unspecific pyramidal cells are shown in blue (30 cells from each HC). ***b***, Single-trial averaged firing rate (15 ms bins) of the entire stimulated pyramidal population (red) and a local subpopulation (MC_0_, orange) over time.

The network displays a ground-state (see first second of activity in Fig. 3*a*,*b*) characterized by low-rate, irregular, asynchronous firing of pyramidal cells. Local basket cells often spike together but do not synchronize firing activity globally. The targeted stimulation of one MC in each HC increases firing in the stimulated population (red), which leads to rapid bursting of local basket cells, which in turn inhibit all neurons in their respective HC, resulting in lower firing of nonspecific (blue) cells. More generally, the network counterbalances increased activity in some MCs by a decrease of activity in neighboring populations. The fast feedback inhibition also leads to fast local burst cycles during high-rate activity. This can be seen best in the firing rate of the orange population, a stimulus-specific MC, local to the first HC.

The evoked firing rate in the targeted population drops over the course of the prolonged stimulation due to accumulating neural adaptation and synaptic depression. This temporarily silences the population after stimulation offset (see Fig. 3*a*, red population between 2 and 3 s), as opposed to the nonstimulated pyramidal population (blue), which however exhibits a significant reduction in firing rate both during and after the stimulus.

Approximately 1 s after stimulus offset, we observe brief, spontaneous reactivations, as the originally stimulated MCs start to fire together again. Locally, these reactivations are γ oscillation bursts, as can be seen in the firing of an isolated stimulus-selective MC (Fig. 3*b*, orange trace). Stimulus-selective pyramidal cells inside each HC (and to a weaker extent neighboring HCs) synchronize due to fast feedback-inhibition and short-connection delays in excitatory associative connections. The pattern-specific firing rate increase (of ∼25 Hz) during attractor reactivations is rather stable for the duration of the reactivation, indicating that most HCs are out of phase with each other with respect to the fast oscillation (some spatially limited degree of transient spike synchronization can be seen in bands of firing that cross different HC during a fast oscillation cycle). After ∼120 ms, the attractor self-terminates due to synaptic depression and neural adaptation. When these have decayed back to lower levels ∼1 s later, we observe further spontaneous reactivations, resulting in a pattern of repeated spontaneous attractor reactivations in discrete oscillatory bursts, very similar to Lundqvist et al. (2011), but based on a different mechanism (Hebbian STP instead of facilitation) and as a result of new learning.

The exact onset and length of reactivations/bursts are somewhat random. Depending on adaptation and synaptic depression variables, attractor activations can stretch out much longer. Some tunings can produce strictly persistent activity (see Persistent activity). Intratrial and intertrial averaging reveals a reliable increase in the global firing rate of the stimulus-specific population from 1.3 Hz (irregular firing) before the stimulation to 2.7 Hz after the stimulation (periodic γ bursting).

### Demo 2: multi-item WM: list learning without intermittent replay

We now attempt to learn more than one item using the same model as before. The goal of this demonstration is mainly to introduce the reader to multi-item memory dynamics in the model, as this will facilitate understanding of the more complicated Simulation Study 1, which then aims to recreate actual experimental findings.

**Figure 4.**
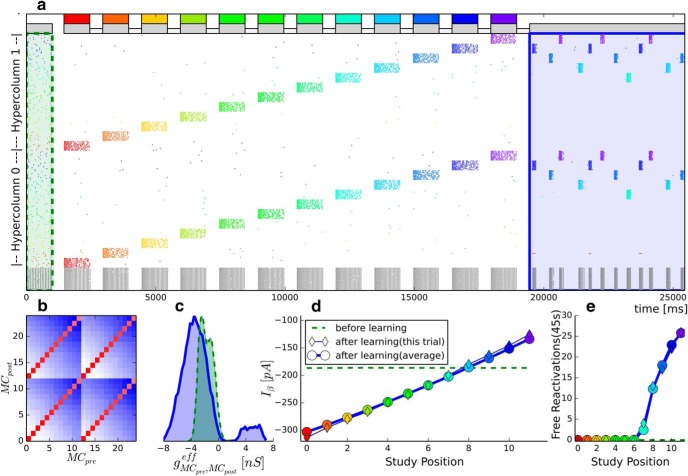
Multi-item WM without intermittent replay. ***b–e***, Multitrial averages are computed over 200 simulations. ***a***, Spike raster of a simulation with 12 training patterns. The raster shows subsampled activity of two HCs, with neurons subgrouped by their respective MC (subsampling 10 pyramidal neurons per MC). Gray represents basket cells. Pyramidal cells are colored according to their pattern selectivity. The last second of the initial 20 s ground state is shown (shaded in green). The first 6 s of the free recall period is shown (shaded in blue). Gray and colored bars on top of the spike raster represent unspecific background input and targeted stimulation. ***b***, Effective mean conductance between pyramidal cells in MC_pre_ and MD_post_. Red represents strong excitatory connections. Blue represents inhibition. MC_0_-MC_11_ are subpopulations of HC_0_. MC_12_-MC_23_ belong to HC_1_. ***c***, Distribution of effective mean synaptic conductances as measured before (shaded green) and after (shaded blue) learning. ***d***, The BCPNN learning rule changes the intrinsic excitability current (see Eq. 10). This plot represents the average bias current for neurons belonging to the different patterns, as denoted by their color. ***e***, Number of pattern reactivations recorded over 45 s of free recall (the spike raster shows only the first 6 s). The legend is the same as in ***d***.

For the first 20 s (Fig. 4*a*, green shaded area), the network is in ground state (see Stimulation protocol). The uncorrelated background, and evoked irregular spiking distributes the initial synaptic weights/conductances (as computed from the *P* traces, see Eq. 11), membrane voltages and other internal network states, such as adaptation or depression. Pyramidal cell activity periodically triggers local basket cells, as in Demo 1. The causal nature of this short-lived feedback inhibition, albeit not nearly as strong as during pattern stimulation or reactivation, is learned by the plastic synapses. AMPA, NMDA, and GABA receptors establish a functional associative network. To show the combined effects of synaptic plasticity in a compact way, we read out the strength of the learned excitation and disynaptic inhibition and combine them to compute an effective mean conductance between pyramidal cells in different MCs, *g_MC_pre_,MC_post__^eff^* (Fig. 4*b*,*c*). Over the course of the first 20 s, the distribution of these values shifts from its initialization at zero into the negative (Fig. 4*c*, green shaded area).

After this initialization, we successively stimulate the network with 12 patterns for 1 s each, with interstimulus intervals of 500 ms. In this particular demonstration, we slightly lower the background rate (−24%) just below levels that would allow for attractor convergence, such that the networks ability to freely recall items is impaired. This does not disable memory per se (which is stored in highly plastic synaptic connections rather than persistent activity), but mostly quiets the network between stimuli. The reduction of unspecific background activity can be thought of as the result of a competing neural event and may correspond to an experimental WM study with a distractor task (Tzeng, 1973), where attention is diverted to abolish active maintenance. What this means for the model will become clearer when we contrast Demo 2 against the two simulation studies later on. Over the course of the learning episode (Fig. 4*a*, ending by the vertical blue line), the network encodes statistical properties of the structured input, as reflected by strong associative weights between neurons in coactivated MCs (Fig. 4*b*, red; and corresponding values near 5 nS in the blue shaded conductance distribution Fig. 4*c*) and inhibitory connections toward neurons in MCs participating in other patterns (Fig. 4*b*, blue, and corresponding values near −4 nS in the blue shaded conductance distribution Fig. 4*c*). Patterns are sparse, so the network learns more disynaptic inhibitory than excitatory connections.

The mean intrinsic excitability current is stable near −180 pA after the initial unstructured input and has approximately the same value for all pyramidal neurons (Fig. 4*d*, green dashed line), as they were recently active equally. After stimulation, we observe that recently active neurons feature a less negative bias current than neurons that have been silent for a long time. This leads to an almost linear relationship between how recently a pattern has been trained and the intrinsic excitability of its pyramidal member neurons.

Finally, during the free recall phase (6 s of which are shown in Fig. 4*a*, blue shaded area), we evaluate memory performance on the basis of autonomous replay for 45 s. For this, we raise the background activity to its original level again and track the autonomous attractor reactivations. Reactivations of recently trained attractors predominate (Fig. 4*e*). Over the course of 45 s, the network freely recalls only the last 5 patterns. All earlier patterns are not reactivated in free recall. They can nearly always reactivate in cued recall, however. We will demonstrate this dramatic difference between cued and free recall in Simulation Study 2.

### Demo 3: PSPs in a loaded cortical attractor memory

A critical question for attractor networks is what the necessary conditions are for attractor activity in modular cortical networks. Among these are requirements on the number of active inputs to a pyramidal neuron participating in an active attractor, the magnitudes of PSPs onto that neuron, and their temporal coordination. To help address this question and validate our model, we briefly take a look at the PSPs that stabilize the cortical attractors in our model.

After learning in Demo 2, pyramidal neurons typically receive active excitatory input from on average 96 presynaptic pyramidal cells (see Network model and connectivity) in the same attractor. To look at the PSPs underlying successful attractor activation in our model, we recorded the membrane potential of a neuron participating in the last learned memory, which could always be reactivated in free recall, so we knew that its recurrent connectivity was strong enough for reactivation.

**Figure 5.**
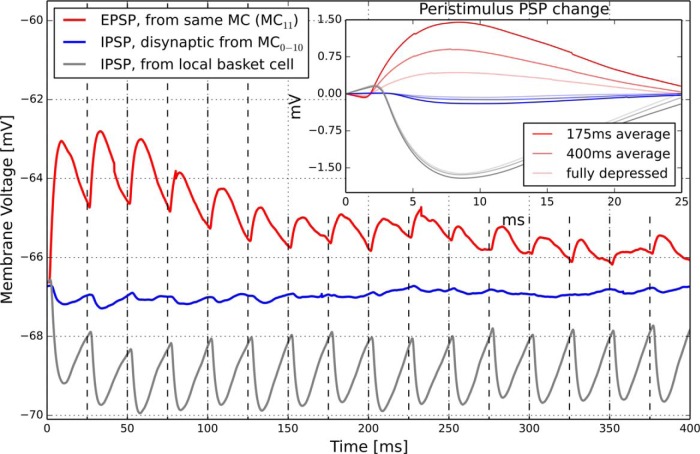
Average PSP of a pyramidal neuron under a 40 Hz presynaptic spike train from one of the following: a presynaptic neuron also in the same MC (red), a presynaptic neuron in another MC in the same HC (blue), or a local basket cell (gray). Synapses may depress, so the peristimulus PSP magnitude (Inset) depends on the duration of the 40 Hz input (25 ms interspike interval). At the ground state near −67 mV, EPSP (red) amplitudes are initially large but quickly depress after just a few spikes. At ground state, disynaptic inhibition amplitude (blue) is only −0.2 mV. Strong inhibition incurred from a presynaptic basket cells (gray) does not depress (see Connectivity).

In three separate scenarios, we excited one particular kind of presynaptic neuron to spike at 40 Hz. Because of the ongoing background input and network activity, postsynaptic activity fluctuates between reset voltage and spiking activity, so we obtained isolated PSP by averaging several hundred recorded postsynaptic traces. We know that attractors are typically active <200 ms, so Figure 5 (inset) implies that a peristimulus EPSP of 1.5 mV magnitude is apparently sufficient for attractor activity in our model. Thomson et al. (2002) measured EPSPs of local layer 2/3 pyramidal-pyramidal cell connections in rat cortex at 1.7 ± 1.3 mV, whereas long range connections have been estimated to be one order of magnitude weaker (Gilbert et al., 1990). Because the temporal coordination of EPSPs is crucial in attractor operation, it is worthwhile pointing out that intraminicolumnar connection delays in our model (1.5 ± 0.23 ms, see Spatial organization) are very similar to those reported by Thomson et al. (2002) between layer 2/3 pyramidal neurons (1.5 ± 0.3 ms). Furthermore, our model's connection probability (*p_PP_* = 0.2, see Connectivity) is only slightly lower than the 0.25 connection probability between local layer 2/3 pyramidals, as reported by Thomson et al. (2002).

Our disynaptic IPSPs (blue) show an amplitude of just 0.2 mV at a membrane potential of −66.8 mV. As the postsynaptic neuron comes closer to the firing threshold, this amplitude will double to ∼0.4 mV (data not shown). Thomson et al. (2002) measured interneuron-pyramidal IPSPs at such higher membrane voltage between −55 mV and −65 mV and recorded amplitudes of 0.65 ± 0.44 mV.

In addition to these regular spiking nonpyramidal interneurons, there are also local, horizontally projecting basket cells. The most effective inhibition in our model comes from these cells, featuring an average constant IPSP magnitude of 1.61 mV, as their connections are not depressing. Thomson et al. (1996) reported corresponding IPSP magnitudes of 1.65 ± 0.32 mV in rat cortex. Basket cells are relatively few, and their inhibition needs to be strong enough to counter active attractor EPSPs, to generate γ oscillations.

### Simulation Study 1: multi-item WM: list learning with intermittent replay

**Figure 6.**
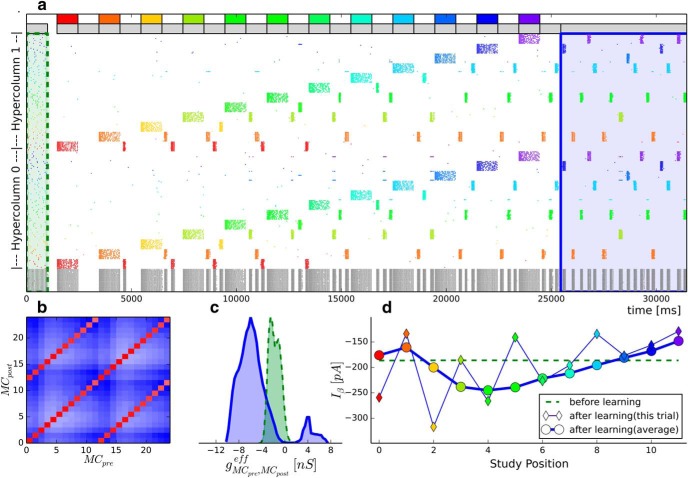
Multi-item WM with intermittent replay. Similar to Figure 5 with the following differences. ***a***, Background activity now causes autonomously generated random reactivations of previously learned patterns in the interstimulus interval. As these reactivations are learned by the network, they actively maintain memories. ***a***, Spike raster of a simulation with 12 training patterns. The raster shows sub-sampled activity of two HCs, with neurons subgrouped by their respective MC (subsampling 10 pyramidal neurons per MC). Gray represents basket cells. Pyramidal cells are colored according to their pattern selectivity. The last second of the initial 20 second ground state is shown (shaded in green). The first six 6 s of the free recall period is shown (shaded in blue). Gray and colored bars on top of the spike raster represent unspecific background input and targeted stimulation. ***b***, Effective mean conductance between pyramidal cells in and . Red represents strong excitatory connections. Blue represents inhibition. MC_0_-MC_11_ are subpopulations of HC_0_, while MC_12_-MC_23_ belong to HC_1_. ***c***, Distribution of effective mean synaptic conductances as measured before (shaded green) and after (shaded blue) learning. ***d***, The BCPNN learning rule changes the intrinsic excitability current (see Eq. 10). This plot represents the average bias current for neurons belonging to the different patterns, as denoted by their color.

**Figure 7.**
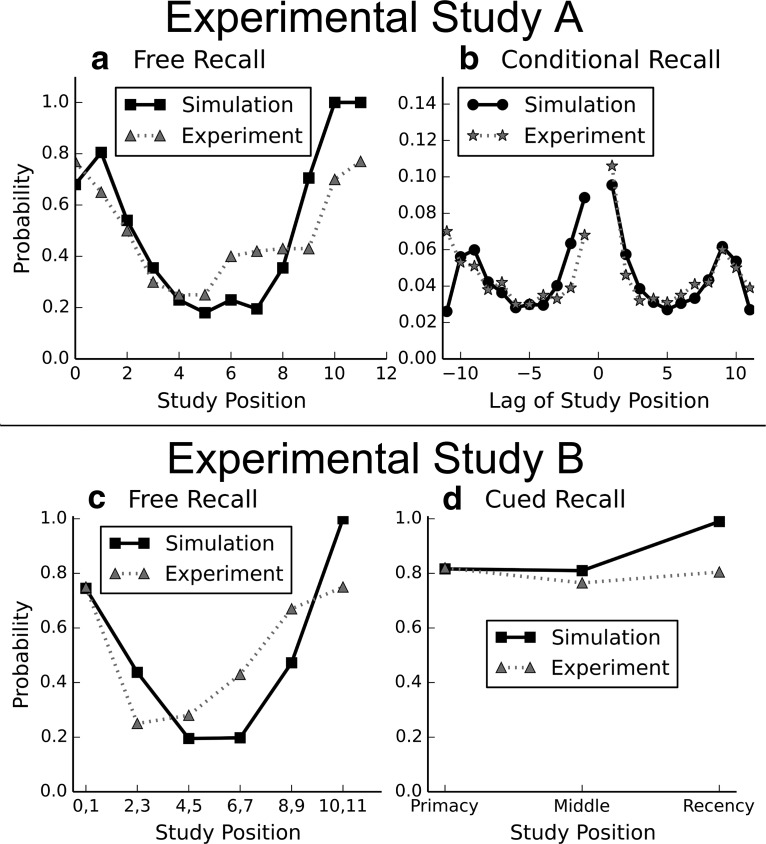
Comparing memory performance metrics between simulation (200 simulation average) and human performance in two word list learning tasks with free and cued recall. ***a***, ***b***, Experimental setup (see Behavioral data comparisons: Experimental Study A). ***c***, ***d***, Experimental setup (see Behavioral data comparisons: Experimental Study B). ***a***, Serial position recall curve, denoting the likelihood of successful free recall (45 s) by the study position. ***b***, Conditional recall probability, as measured by the distribution of study position lag between successively recalled memory items in free recall. ***c***, Serial position recall curve in free recall over 30 s. ***d***, Serial position recall curve in cued recall.

We now use our model to capture the design of the experimental word list learning task described earlier (see Behavioral data comparisons). Similarly to the described Experimental Study A, 12 items were presented at a rate of one item per 2 s with intermediate pauses and a subsequent free recall phase of 45 s (Fig. 6). A crucial difference to the earlier Demo 2 is that we now leave the background activity rate untouched (i.e., no reduction between stimuli), so the network can autonomously reactivate attractors in the interstimulus interval. This leads to very different learning outcomes. Early patterns can now survive and even further strengthen themselves in the network through intermittent reactivations following their initial learning episode. This autogenic process can be described as a form of memory refresh, or short-term memory consolidation, and in the context of words even likened to the phonological loop. It increases the memory capacity and dynamic complexity of the model. Free recall shows higher capacity; 5–8 different patterns can now be recalled (>6 in the multitrial average). Which patterns these are, is different from trial to trial, but primacy and recency are prominent, as the U-shaped pattern recall probability curve (Fig. 7*a*) shows.

The computational model broadly recreates both the U-shaped serial position recall curve (Fig. 7*a*), as well as the peculiar Mexican-hat-shaped conditional recall probability (Fig. 7*b*) found in Experimental Study A (Nilsson et al., 1997). Just like subjects of the study, the model shows a slight propensity for sequential replay (as indicated by increased transition probabilities with lag ±1). Transition probabilities also increase for extreme lags because the U-shaped serial position curve makes transitions between early and late items (i.e., large absolute lags) more likely than transitions to and from middle position items.

### Memory consolidation as a competitive process

A key observation to understand the performance of the word list learning model is that the autogenic memory refresh is a competitive process. The network is highly plastic, so intermittent reactivations are necessary for successful maintenance of any pattern that was not stimulated recently. Early patterns face less competition, so it is likely that they can establish themselves in the network through repeated reactivations, which in turn promotes further reactivations and eventual free recall. Late patterns do not even need to reactivate before successful recall. Middle patterns fare worst because they need a few reactivations to survive until the free recall testing but face stiff competition from earlier patterns with often higher excitability. At most, three or four attractors can reactivate in the short time between externally driven pattern stimulations. The mere existence of some reactivations is not sufficient, however. In the above spike raster (Fig. 6*a*), we can see that pattern 0 (red), for example, reactivates a number of times but is ultimately not recalled in the end. The pattern eventually loses the competition for further reactivations against other patterns long before the eventual free recall episode. Further, it is not necessary that a pattern reactivate in every stimulation pause after it is initially learned. In the shown trial, patterns 1 (orange) and 5 (green) skip several opportunities for reactivations, and pattern 3 (olive green) is silent for 6 s directly preceding the free recall episode, yet all these patterns are successfully retrieved during the first 6 s of free recall. More generally, recalled patterns have usually been either stimulated or autonomously reactivated within the last 8 s of the free recall period.

The mean firing rate of eventually recalled patterns in our model increases from 1.3 Hz at baseline to 2.7 Hz after learning, although not successfully recalled patterns show a strong reduction in overall spiking activity. In a review of experimental findings, Shafi et al. (2007) concluded that the overall increase in firing rate of stimulus-selective cells is generally small (<5 Hz), such as a 1.78 Hz increase (54% above baseline) in delay-activated cells in PFC during a visual single-item delayed match to sample task with an 18 s delay following the stimulus (Quintana et al., 1988).

### Simulation Study 2: cued recall in word list learning

Some memory patterns, although not recalled spontaneously in free recall, are nevertheless still kept in memory and can be retrieved by stimulating with a cue. There are several possible ways to test cued recall, also called pattern completion in the context of attractor memory models. In keeping with the idea of modeling a 12-word list learning task, we can compare our model performance against data from Gershberg and Shimamura (1994) (see Behavioral data comparisons, Experimental Study B). For a direct performance comparison, we adopt the experimental study's stimulation timing (i.e., sequential training, 12 words, one word per 2 s) and metrics for free recall (i.e., 30 s free recall, averaging free recall performance of sequential pairs of learned words) and cued recall (i.e., testing each pattern individually with a 5 s recall time limit after each cue consisting of half-patterns, and a three part division of serial position for recall curve plotting).

Notably, cued recall (Fig. 7*c*) is much more likely to retrieve the pattern than free recall (Fig. 7*d*) in both experiment and model. Weak middle position patterns that have a free recall probability of ∼20%-30% can be recalled using an appropriate cue ∼80% of the time. There seems to be a recency effect in cued recall in the model; but as Gershberg and Shimamura (1994) already pointed out, the ceiling effect distorts serial position analysis when the task is too easy. A deeper analysis of a more challenging task is, however, out of the scope of this paper.

## Discussion

We set out to show that Hebbian STP can be used to build a functional cortical WM. Our model supports this and also the hypothesis that WM encoding, maintenance and reactivation manifests in discrete oscillatory bursts rather than persistent activity. Contrary to earlier models based on facilitation, our model is capable of encoding novel items and goes further in bridging the scales of neuroscientific inquiry from synapse to behavior from a modeling perspective. Apart from this crucial difference, the model is closely related to the one by Lundqvist et al. (2011), explaining recall, active maintenance of multiple items, and serial position effects. Our model quantitatively matches selected cognitive memory studies of serial position effects, conditional recall, free recall, and cued recall, and reproduces results from a previous non-spiking model of word list learning (Lansner et al., 2013). It is worth noting that Hebbian plasticity does not exclude other synaptic and neural plasticity mechanisms (facilitation, augmentation, dendritic voltage bistability, etc.) that may well act in parallel.

In the following, we will briefly discuss the experimental support for fast Hebbian plasticity, the model's relationship to the persistent activity hypothesis, and other ideas about WM activity, the serial position curve, and highlight the dynamic memory structures created by the plasticity mechanism. Last, we discuss the electrophysiological dynamics of attractor activations during WM maintenance.

### Experimental support for fast Hebbian synaptic plasticity

A main argument against Hebbian forms of synaptic WM has been that LTP does not induce as a result of only a brief paired activation and further takes quite some time to express in the form of a significant conductance change. Once formed, it is also long-lived, which is incompatible with a volatile memory, such as WM. In the past few years, however, different early forms of LTP, such as E-LTP (Park et al., 2014) and STP (Erickson et al., 2010; Volianskis et al., 2015), have been characterized experimentally and proposed as candidates for a synaptic WM. This includes observations that fast STP can last for 6 h when there are no or very few presynaptic (read-out) spikes (Volianskis et al., 2015), suggesting activity-, rather than time-dependent, decay mechanisms for memory. A full review of fast Hebbian synaptic plasticity is out of the scope of this article, but we will here provide some pointers to relevant experimental results.

E-LTP, is NMDA-dependent, but independent of protein synthesis. Candidate mechanisms include increased presynaptic transmitter release, AMPAR phosphorylation by CaM-CaMKII, receptor insertions from intracellular cytosol, or translocation from perisynaptic locations. Computational modeling suggests that considerable effective synaptic conductance change can happen already some seconds after the induction signal (He et al., 2015). Stimuli too weak to induce LTP can still induce NMDAR- and GluR1-dependent Hebbian STP (Erickson et al., 2010). Several closely spaced stimuli in the test pathway, forming a single brief burst, were sufficient to induce STP, which then decayed with two time constants: a fast component (1.6 ± 0.26 min) and a slower one (19 ± 6.6 min). Potassium from postsynaptic NMDAR activation has been proposed as a retrograde messenger for presynaptic STP induction (Park et al., 2014), also implying a Hebbian learning rule. Further investigation is required to further elucidate and dissect these phenomena.

### Persistent activity

**Figure 8.**
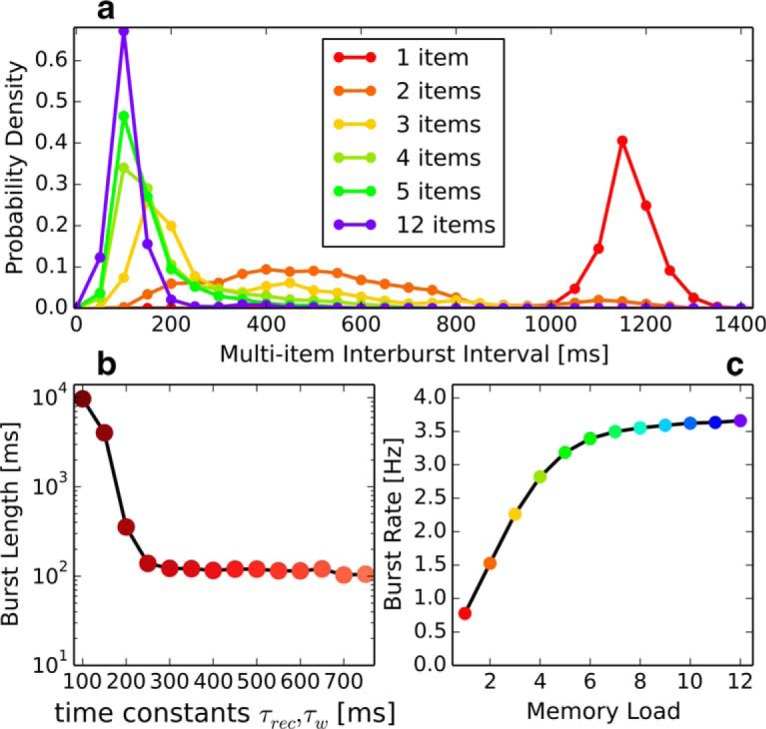
Reactivation statistics recorded over 10^4^ ms delay periods in 100 trials. ***a***, Probability density of interburst intervals as a function of memory load, denoting the number of trained patterns (not necessarily recalled). ***b***, Mean burst length of a single-item memory as a function of covaried τ_rec_, τ_w_, which denote the time constants of synaptic depression and neural adaptation, respectively. ***c***, γ burst rate as a function of memory load.

We demonstrate that WM encoding and maintenance can be mostly silent with reactivations manifested in discrete oscillatory bursts. In free recall testing, we find that memories can be reactivated after many seconds of silence, and even longer in cued recall. Such long silence contradicts a strict interpretation of the persistent activity hypothesis but is compatible with experimental findings that an active memory trace may not be necessary for short-term retention (LaRocque et al., 2013). This does not mean that there is no memory-related increase in overall activity of memory-specific cells in our model, however. Single- and multi-item memory scenarios generally exhibit an increase of overall activity for successfully maintained WM patterns. The precise temporal onset and interval between brief reactivation-related bursts in our model are somewhat random (Fig. 8*a*). Averaging large-binned (e.g., 500 ms) spike counts over several trials, as commonly seen in early experimental studies, would hide bursts and their modulation, thus showing a seemingly stable and “persistent” activity of successfully recalled items during maintenance, elevated by a few Hz.

In a review of experimental findings, Shafi et al. (2007) concluded that individual cells bridging a multisecond delay are exceptionally rare, that the overall increase in firing rate of stimulus-selective cells is generally small (<5 Hz), especially in PFC, and that “… stable persistent activity during working memory is often an artifact resulting from averaging away intratrial variability ….” Experimental findings repeatedly link information in multi-item WM tasks to discrete oscillatory bursts in the γ band (Honkanen et al., 2015; Lundqvist et al., 2016). This stands in direct contrast to historical findings of (and emphasis on) persistent activity in single-item delayed match to sample tasks and their interpretation (Quintana et al., 1988; Funahashi et al., 1989; Goldman-Rakic, 1995).

It seems likely that WM manifests as multiple forms of activity patterns in the brain, including bursting attractor dynamics, persistent activity, fast neural sequences akin to synfire chains, and phase relationships. Various forms of activity averaging and generalization from single-item retention may confound differing but not necessarily exclusive hypotheses about the mechanisms behind WM. For example, multineuronal sequential firing patterns have been found to coexist with highly irregular firing and attractors in a related model (Herman et al., 2013). Despite its focus on fast Hebbian plasticity, our model is entirely compatible with other maintenance mechanism, such as facilitation (as in the aforementioned model), provided attractors are encoded first. Although our model exhibits finite burst length, it can also be tuned to achieve “stable” persistent attractor dynamics in the single item case (Fig. 8*b*).

### Serial position effect

Repeated findings of robust primacy and recency across different task and sensory modalities (Ward et al., 2005) have made the serial position effect relevant for the overall understanding of memory and inspired early cognitive memory models. Our spiking network implementation succeeds in capturing serial position effects and explains them as result of fast Hebbian plasticity, intrinsic excitability, and an emergent autogenic process of competitive memory consolidation in the interstimulus period. In contrast to common cognitive multistore models (Atkinson and Shiffrin, 1968), our model requires only a single-store/network to account for serial position effects in free recall and increased memory capacity in cued recall. Many factors modulate the shape of the WM serial position curve and deserve future exploration.

Experimental data that could support a causal link between intermittent reactivations and serial position effects remain elusive, but it is worth pointing out that a causal link between discrete replay events in hippocampus and memory consolidation has much support for long-term memory. The amnesic effects of targeted replay interruption via electrical stimulation (Girardeau et al., 2009; Ego-Stengel and Wilson, 2010) suggest that this link is causal, not merely correlational, and has previously been modeled as such by the authors (Fiebig and Lansner, 2014).

### Dynamic memory structures

We do not rely on any preshaped synaptic structures (except for local basket cell circuits that define HCs). Structured input can reshape connectivity at any point in time. Newly formed attractors are immediately subject to known associative memory dynamics, such as pattern completion, rivalry, perceptual blink, reactivation dynamics with fast oscillation bursting, etc.

Learned associative weights in our model remain plastic throughout. Because learned weights capture the statistical properties of recent firing activity and generate activity with similar statistical properties, we do not need to modulate or gate plasticity to guarantee stable weights and activity. Targeted modulation (most notably via dopamine) is still a likely scenario, however, and could be incorporated into the model (see factor κ in Eq. 9) to facilitate processes, such as attentional gating and novelty detection.

### Electrophysiological dynamics of attractor activations

Our findings supports the hypothesis that WM maintenance and reactivation are manifest in discrete oscillatory bursts rather than sustained activity, in agreement with recent experimental work (Lundqvist et al., 2016). Global attractor activations (mean length 120 ms) are composed of near-simultaneous local γ burst cycles (Fig. 3*b*) that are out-of-phase with each other. Pyramidal cells in local MCs spike synchronously 3–5 times over a window of ∼100 ms, whereas Lundqvist et al. (2016) reported five fast cycles per γ burst with a combined length of 76 ms. Interestingly, just such discrete γ bursts were found to be optimal for the induction and maintenance of STP in experiments (Park et al., 2014).

Attractor lifetime and the interval between activations in our model are highly dependent on the magnitudes and time constants for spike-triggered synaptic depression and neural adaptation (Fig. 8*c*). Limited attractor lifetime allows for concurrent encoding of multiple memory items and reduced interference between them. This may also reduce total energy expenditure when compared with persistent activity, as action potentials and their postsynaptic effects account for ∼80% of the estimated energy budget of the brain (Attwell and Laughlin, 2001).

Because of parallel encoding, we observe a load-dependent increase of discrete burst events up to a capacity limit of 5 or 6 items. Mean burst rates increases from ∼1 burst/s for the single-item memory to 3.8 bursts/s in the 12-item condition (Fig. 8*c*). We predict that such an increase is proportional to the number of items when the load is low, but quickly saturates at the capacity limit of 5 or 6 items (Fig. 8*c*). Similar load-dependent proliferations of brief γ burst events have been predicted by a related model (Lundqvist et al., 2011) and found in experiments (Axmacher et al., 2007; Lundqvist et al., 2016). For example, Lundqvist et al. (2016) recorded a load-dependent increase in mean burst rate from 3 to 4.5 bursts/s (Pawel Herman, personal communication) when load was increased from 2 to 3 items. Our model predicts a very broad distribution of interburst intervals at this exact memory load (Fig. 8*a*), providing a possible explanation for their failure to isolate a slow burst-rate modulating frequency. In confirmation of model observations made by Lundqvist et al. (2010), we observe that the overall network size plays an important role for the stability of the fast local oscillatory regimen. Large and diverse delays of long-range connections establish out-of-phase excitation, which is critical to reactivate populations after triggered local feedback inhibition. This is why we simulated a subsampled cortical patch instead of a more complete, but spatially smaller area.

Attractor size and strength are also important for the stability of attractors. We show that Hebbian STP yields plausible EPSP and IPSP magnitudes following attractor learning in the model. The number of learned excitatory inputs onto a pyramidal neuron in an attractor is ∼100. In conjunction with biologically plausible PSPs, firing rates, local connectivity, and connection delays in our model, this constitutes a testable prediction for biological cortex. The number of excitatory incoming connections from the same attractor onto pyramidal neurons in layer 2/3 should be ∼100 to give a synaptic current sufficient to stabilize an attractor state.
